# Autonomy, Resilience and Life Satisfaction among Badminton Paralympians

**DOI:** 10.21315/mjms2024.31.2.15

**Published:** 2024-04-23

**Authors:** Poh Li Lau, Siew Li Goh, Emily Kui Ling Lau, Kuan Garry, Yee Cheng Kueh, Ngan Ling Wong

**Affiliations:** 1 Department of Educational Psychology and Counselling, Faculty of Education, Universiti Malaya, Kuala Lumpur, Malaysia; 2 Sports and Exercise Medicine Research and Education Centre, Centre for Epidemiology and Evidence-Based Practice, Faculty of Medicine, Universiti Malaya, Kuala Lumpur, Malaysia; 3 Department of English Language, Faculty of Languages and Linguistics, Universiti Malaya, Kuala Lumpur, Malaysia; 4 Exercise and Sports Science Programme, School of Health Sciences, Universiti Sains Malaysia, Kelantan, Malaysia; 5 Biostatistics and Research Methodology Unit, School of Medical Sciences, Universiti Sains Malaysia, Kelantan, Malaysia; 6 Department of Asian and European Languages, Faculty of Languages and Linguistics, Universiti Malaya, Kuala Lumpur, Malaysia

**Keywords:** athletes, autonomy, disabled, life satisfaction, motivation, resilience

## Abstract

**Background:**

People with disabilities are marginalised in mainstream culture and they also experience increased restrictions in a variety of areas, such as sport. The barriers they encounter may adversely affect their life satisfaction, especially if they have a low perceived sense of autonomy and resilience. The purpose of this study was to investigate the relationship between autonomy, resilience and life satisfaction in para-badminton athletes and the mediating role of resilience in the relationship between autonomy and life satisfaction.

**Methods:**

Data were collected from 137 para-badminton athletes (male: 65.0% and female: 35.0%). Self-reported measures were used to assess the participants’ autonomy, resilience and life satisfaction levels.

**Results:**

A structural equation model analysis was performed; the model had sufficient fit indices (comparative fit index [CFI] = 0.94, root mean square error of approximation [RMSEA] = 0.06, standardised root mean square residual [SRMR] = 0.07). The findings showed that autonomy had a significant effect on resilience (*β* = 0.32, *P* = 0.001). Further, resilience had a significant effect on life satisfaction (*β* = 0.19, *P* = 0.011) and significantly mediated the effect of autonomy on life satisfaction (*β* = 021, *P* = 0.033).

**Conclusion:**

This study revealed that autonomy fosters resilience among athletes, in turn allowing them to achieve greater life satisfaction. Therefore, society and sport communities should actively seek ways to improve the autonomy and resilience levels of athletes with disabilities.

## Introduction

Around 1.5 billion individuals worldwide have a disability—that is, a long-term physical, intellectual, mental or sensory restriction that makes it difficult for them to participate fully and fairly in society (1). Disability has traditionally been medicalised as a sickness that must be treated; however, it is now viewed as a continuum of the human condition that may be productive, creative, affirming and pleasant (1). Sports for people with disabilities are physical activities for those who, as a result of their illness, injury or disability, find it difficult to participate in generally accessible sports and physical activities that require specialised abilities and unique applications (2), such as specialised equipment, tools and techniques.

Golf was among the earliest organised sports for people with disabilities and there are now many other sporting events for disabled people all over the world (3). Para-badminton was initially introduced to the world in 1995 at the Stoke Mandeville Games and the number of countries participating in para-badminton games and competitions has increased dramatically since then. Ever since Asian countries began participating in international para-badminton games, the number of players has more than doubled and the level of competition has drastically improved in some classes (3). The Badminton World Federation (BWF) currently oversees para-badminton on a global scale.

The health of people living with disabilities (PLWD) is poorer than that of the general population (4). This may be because PLWD are more likely to suffer injuries, develop chronic non-communicable diseases and experience age-related health issues early in life (1). Life satisfaction is one of the most important measures of health, quality of life and well-being. It refers to the global cognitive self-evaluation of a person’s whole life, which includes a wide range of activities (5). Notably, research studies have shown that disabled and non-disabled people experience different levels of general life satisfaction (5). Compared to the general population, PLWD are likely to report high quality of life and life satisfaction levels, such as inclusivity, access to support services, and personal resilience and coping skills (6). Furthermore, Schwartz et al. (7) stated that personal standards, values and attitudes regarding health-related quality of life may change over time and vary from person to person, resulting in ambiguous or inconsistent outcomes. It is yet unclear what causes this large diversity in life satisfaction levels among individuals with disabilities.

Team sport offers greater psychological benefits than physical health benefits alone (8). Prior research found that athletes with intellectual disabilities who participated in team sporting events expressed better feelings of striving and belonging (9). Although numerous studies have focused on how sport involvement benefits the quality of life of PLWD, there is limited research involving the application and testing of a theoretical model to examine the motivations and drivers of these positive results (10). According to Deci and Ryan (11), autonomy support is essential for enhancing an individual’s self-determination and, in turn, for promoting other favourable health outcomes throughout one’s life. Several studies have shown that autonomy support increases life satisfaction among athletes in the general population (12, 13). In addition, Komenda et al. (10) found an association between an autonomy-supportive sport climate and life satisfaction, mediated by competence.

Since having a disability is directly related to agitation in difficult situations, which is marked by unpleasant emotions and mental struggles, an essential component in the life of the individual with a disability is resilience—that is, acceptance of the limitations brought on by disability and the challenges resulting from it (14). Resilience increases mental power in impaired athletes, allowing them to deal with future stress and perform a variety of tasks (15). For example, it was reported that wheelchair basketball players with high levels of resilience were the most active in the sport and had the highest quality of life (16). Furthermore, for paralympic athletes in athletics and swimming, resilience appears to be an intervening component of both autonomous (intrinsic, identified regulation) and controlled (introjected, external regulation) motivation (14).

Autonomous motivation and resilience are important psychological factors that help paralympic athletes adapt to and overcome stressful demands, thus improving their life satisfaction (17). In the context of paralympic sport, examining the relationship between resilience and motivation is essential because these factors are very important for the life satisfaction and quality of life of athletes, and few studies have investigated the relationship between resilience and motivation in this context (14). To our knowledge, no research has been conducted on the relationships between autonomy, resilience and life satisfaction among para-badminton athletes. Therefore, the aim of the current study was to investigate the relationship between autonomy and life satisfaction among para-badminton athletes as well as the mediating role of resilience in said relationship.

## Methods

### Sample Size Estimation

According to Kline (18), the ideal sample size in studies where structural equation modelling (SEM) was used is about 200 cases; however, 100 cases can be sufficient when a relatively simple model is being evaluated. In the present study, given that the SEM model is relatively simple with only three latent variables, the sample size was set to be 100. The adjusted sample size was estimated to be 143 after adding a 30% dropout rate (100/0.7) to account for missing values and incorrect data entry.

### Participants

A total of 143 anonymous responses were received, of which 6 (4.2%) had some missing values and 137 (95.8%) had complete responses. European players were best represented (40%), followed by Asians (30%). The remaining responses originated from other continents, such as America and Africa.

### Measures

The revised Brief Resilience Scale (BRS: 3-items) was used to measure the participant’s ability to deal with stress or issues and bounce back from hard times (19). Participants in the study were asked to answer the three BRS questions on a 5-point Likert-type scale ranging from 1 (strongly disagree) to 5 (strongly agree), such as “I have a hard time getting through stressful events.” All three items were converted to positive score for this study and a higher score indicates a higher level of resilience. The internal consistency for BRS was good, with a Cronbach’s alpha of 0.85 (19). Based on Cronbach’s alpha, the internal consistency of the three items’ total score in this study was 0.75.

The Satisfaction with Life Scale (SWLS) was used to measure the participant’s global life satisfaction (20). Participants in the study were asked to rate the extent to which they agreed with each question on a 7-point Likert-type scale ranging from 1 (strongly disagree) to 7 (strongly agree), such as “In most ways, my life is close to my ideal.” The scale consisted of five items and a higher score indicates a higher level of satisfaction. The internal consistency for SWLS was good, with a Cronbach’s alpha of 0.87 (20). Based on Cronbach’s alpha, the internal consistency of the five items’ total score in this study was 0.81.

The three items of the WHO Quality of Life (WHOQOL)-Disability Group (Aut: 3-items) were used to measure the participant’s autonomy (21). The participants in the present study were asked to rate how they felt about themselves over certain aspects of their lives on a 5-point Likert-type scale ranging from 1 (not at all) to 5 (totally), such as “Do you feel in control of your life?” A higher score indicates a higher level of autonomy. The Aut’s internal consistency was good, with a Cronbach’s alpha of 0.76 (22). Based on Cronbach’s alpha, the internal consistency of the three items’ total score in this study was 0.72.

### Sampling and Data Collection

The targeted population included all professional para-badminton athletes. The convenience sampling technique was used to collect data from those who agreed to participate voluntarily in the study. The data was collected between February 2020 and August of 2020. The study was an online, cross-sectional survey across different regions of the world using self-reported questionnaires prepared in English. The BWF distributed study questionnaires to its member associations along with a cover letter outlining the purpose of the study and a privacy declaration. For athletes who were not familiar with the English language, coaches or trainers were encouraged to act as interpreters. This is to avoid bias that could come from different ways of interpreting the questions.

### Statistical Analysis

A descriptive analysis was performed to describe the demographic characteristics of the participants. The results were presented as frequencies and percentages. SEM was performed to determine the effect of autonomy on life satisfaction and the mediating effect of resilience in the relationship between autonomy and life satisfaction using Mplus 7.2. Following the data analytic plan, an analysis using bias-corrected bootstrapping was performed. In the analysis, 5,000 bootstrap samples were generated using random replacement samples. In this research, the multiple linear regression (MLR) is employed since its parameter estimates are unaffected by the normality of the data distribution (23). Hu and Bentler (24) recommended fit indices and cut-off point values were as follows: i) non-normed fit index (NNFI) of 0.96 or higher and standardised root mean square residual (SRMR) of 0.09 or lower; ii) root mean square error of approximation (RMSEA) of 0.06 or lower and a SRMR of 0.09 or lower; iii) comparative fit index (CFI) of 0.96 or higher and a SRMR of 0.09. In addition, all scales were tested using a single-construct measure that calculated the overall score for each scale.

## Results

**Figure 1:**
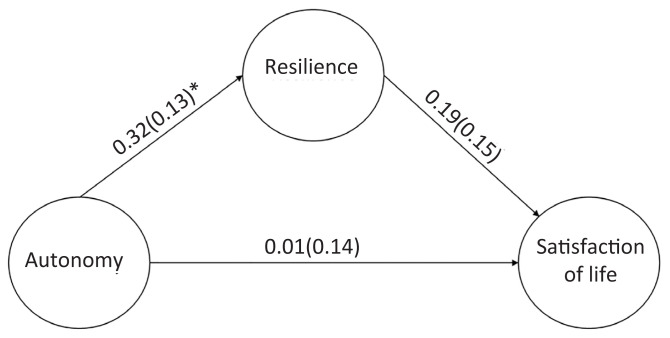
Final structural equation model of the relationships between autonomy, resilience, and life satisfaction and standardised regression coefficients, β (SE)

The study consisted of 89 (65.0%) males and 48 (35.0) females. By classification of disabilities, the wheelchair 1 (WH1) group was the largest group of respondents (27%). More than 60% of the athletes had some source of income, either from fixed-paying jobs, part-time or temporary employment or were self-employed and more than half of these athletes (56.9%) had received tertiary education. Playing years varied widely from above 3 years to more than 10 years (Table 1). The initial hypothesised model achieved the recommended fit indices (Table 2), with autonomy having a significant influence on resilience (*β* = 0.32, *P* = 0.001) and resilience having a significant influence on life satisfaction (*β* = 0.19, *P* = 0.011). Also, resilience significantly mediated the effect of autonomy on life satisfaction (*β* = 021, *P* = 0.033). However, there was no significant direct relationship between autonomy and life satisfaction (*β* = 0.01, *P* = 0.430) (Figure 1).

## Discussion

The findings of this study highlighted the relationships between autonomy, resilience and life satisfaction among a group of para-badminton athletes from different regions of the world. This study is unique, as no other studies have investigated these relationships in the context of para-badminton, although research has been conducted on the relationship between general life satisfaction and various other factors (such as satisfaction with health, family, work and other domains) (25). The United Nations Convention on the Rights of Persons with Disabilities mandates that states parties take adequate steps to enable people with disabilities to participate in sporting activities on an equal basis with others, thereby reflecting the importance of sport for a high quality of life (26).

The present study showed that autonomy has a significant relationship with resilience, and resilience mediates the relationship between autonomy and life satisfaction. This indicates that para-badminton players with high levels of autonomous motivation are better able to deal with challenges, adapt to changes, overcome obstacles and withstand pressure in challenging situations. As a result, they derive more pleasure and life satisfaction from sport. A previous study showed that a positive relationship exists between autonomy and resilience in athletes, which improves their quality of life and well-being (14). Furthermore, according to the self-determination theory, competence and autonomy can strengthen one’s ability to deal with obstacles and tough situations, thus increasing one’s self-determined desire to engage in sport (27).

Unlike previous studies, no direct relationship between autonomous motivation and life satisfaction was found in this study (12). This may be due to the absence of resilience as a mediator between autonomy and life satisfaction in previous studies. Resilience was found to have a significant direct effect on life satisfaction, indicating that athletes’ ability to accept and deal with crises caused by their disabilities can improve their sense of life satisfaction and happiness. Previous studies have also reported a strong relationship between resilience and life satisfaction (14). Sporting activity has been shown to improve one’s resilience and speed up the process of adaptation to disability (28).

The present study had a number of limitations associated with it. First, we used cross-sectional data for this study. Therefore, conclusions about any causal relationships between the variables’ pathways within the current structural model should be drawn with caution. Second, self-reported measures were utilised, which could have led to response bias. Third, the sample size was small, potentially affecting the generalisability of the study findings. It is recommended that future studies use a longitudinal experimental design, as well as larger sample sizes, to investigate these variables.

## Conclusion

The interrelationships between autonomous motivation, resilience and life satisfaction among para-badminton athletes were investigated in the present study. The findings suggest that programmes designed to promote autonomy and resilience can improve athletes’ life satisfaction. Researchers, coaches, and sport psychologists should create programmes that support autonomy and resilience to enhance the life satisfaction and quality of life of athletes with disabilities.

## Figures and Tables

**Table 1:** Demographic characteristics of the participants

Variable	*n* (%)
Gender	
Male	89 (65.0)
Female	48 (35.0)
Continent	
Europe	55 (40.2)
Asia	41 (29.9)
Others	41 (29.9)
Classification	
wheelchair	57 (41.6)
WH1	37 (27.0)
WH2	20 (14.6)
Standing	80 (58.4)
SL3	21 (15.3)
SL4	26 (19.0)
SU5	19 (13.9)
SH6	14 (10.2)
Disability	
Acquired	72 (52.6)
Congenital	65 (47.5)
Income	
Fixed pay	43 (31.4)
Part-time/temporary	40 (29.2)
Self-employed	18 (13.1)
Others	36 (26.3)
Education	
Primary	4 (2.9)
Secondary	52 (38.0)
Tertiary	78 (56.9)
Others/none	3 (2.2)
Competing experience (years)	
< 3	38 (27.8)
3 to < 5	37 (27.0)
5 to < 10	40 (29.2)
≤ 10	22 (16.1)

**Table 2:** Goodness-of-fit indices of the structural equation models (*N* = 137)

Model	df	Scaled χ^2^	RMSEA [95% CI]	CFI	SRMR
Hypothesised structural model	41	60.28	0.06 [0.02, 0.09]	0.94	0.07

Notes: CFI = comparative fit index; SRMR = standardised root mean square residual; RMSEA = root mean square error of approximation; CI = confidence interval
